# Four key challenges in infectious disease modelling using data from multiple sources

**DOI:** 10.1016/j.epidem.2014.09.004

**Published:** 2015-03

**Authors:** Daniela De Angelis, Anne M. Presanis, Paul J. Birrell, Gianpaolo Scalia Tomba, Thomas House

**Affiliations:** a MRC Biostatistics Unit, Cambridge Institute of Public Health, Robinson Way, Cambridge CB2 0SR, UK; b Public Health England, 61 Colindale Avenue, London NW9 5HT, UK; c Department of Mathematics, University of Rome Tor Vergata, Rome, Italy; d Warwick Mathematics Institute, University of Warwick, Coventry CV4 7AL, UK

**Keywords:** Evidence synthesis, Bayesian, Statistical inference, Multiple sources, Epidemics, Complex models

## Abstract

Public health-related decision-making on policies aimed at controlling epidemics is increasingly evidence-based, exploiting multiple sources of data. Policy makers rely on complex models that are required to be robust, realistically approximating epidemics and consistent with all relevant data. Meeting these requirements in a statistically rigorous and defendable manner poses a number of challenging problems. How to weight evidence from different datasets and handle dependence between them, efficiently estimate and critically assess complex models are key challenges that we expound in this paper, using examples from influenza modelling.

## Highlights

• Health decision making increasingly uses models and data from multiple sources.
• Inference on model parameters using a multiplicity of data sources is challenging.
• Key challenges include more thoughtful model specification and criticism.
• Addressing these problems rigorously will require better use of existing tools.
• Challenges in epidemic models may motivate new statistical methods.

## Introduction

Increasingly, there is a perceived need to exploit information from multiple sources in epidemic modelling, ensuring decision-making on public health policies geared to control epidemics is progressively based on as many diverse sources of information as possible (Rutherford et al., 2010) and the use of models (e.g. https://www.gov.uk/government/policy-advisory-groups/joint-committee-on-vaccination-and-immunisation). Policy makers want ‘defendable’ models that not only realistically approximate the phenomenon of interest, but are also, crucially, able to produce outputs consistent with all relevant available data (Rolka et al., 2007; Lipsitch et al., 2011). This requirement, supported by the continued progress in computational power, has encouraged the development of increasingly complex models, which, in turn, require rich arrays of data to guarantee parameter identifiability (Ferguson et al., 2006).

In addition, irrespective of the complexity of the model, modellers are often faced with the task of integrating information from many heterogeneous sources of data. For example, the behaviour of an epidemic in its early stages is described by the parameter *R*_0_, the basic reproductive number. However, equally crucial for the containment of an infectious disease outbreak (Fraser et al., 2004; Powers et al., 2011) is knowledge of the proportion of transmission occurring before the onset of symptoms, *θ*. Population incidence data contain information on *R*_0_, but are uninformative about *θ*. Complementary evidence from ‘challenge’ studies, where the time between infection and symptom onset is measured directly and information is available on the distributions of latent and infectious periods, are needed to estimate *θ*. A comprehensive description of the evolution of an outbreak can only be obtained using data from multiple sources.

**Fig. 1:**
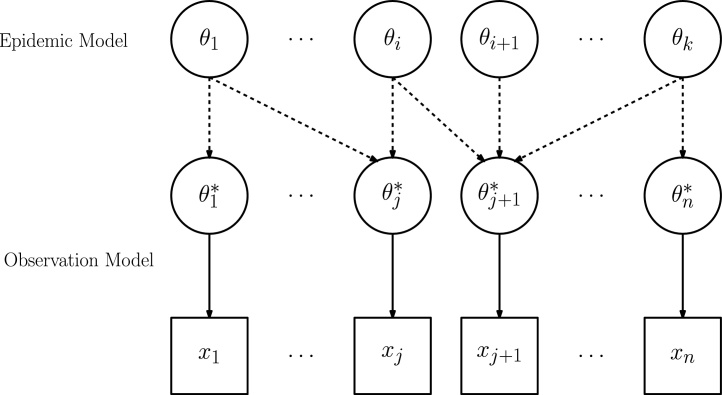
Schematic diagram of how multiple data sources can link into an epidemic model via an observation model(s).

It is, however, not typically the case that there will be a single data source directly informing each relevant parameter. More realistically, there will be a collection of datasets, each of different quality, that will need to be appropriately synthesised to derive the estimates of interest, as illustrated in Fig. 1. Here the epidemic process is modelled in terms of the basic parameters of interest, ***θ*** = {*θ*_1_, …, *θ*_*k*_} and the information from each data source *x*_*j*_, *j* = 1, …, *n*, is expressed as a function of the basic parameters i.e. $\theta_{j}^{*}=f_{j}(\theta )$. The form of this function, whether deterministic or stochastic, defines the relationship of the observation model to the epidemic model. Examples of *f*_*j*_(***θ***) include cases where a data source provides: direct information on a single parameter of interest (i.e. $\theta_{j}^{*}\;=\;\theta_{i}$); biased evidence on ***θ*** (see Section “Model criticism”); simultaneous information on multiple components of ***θ*** or on further nuisance parameters ***ϕ*** (i.e. $\theta_{j}^{*}=f_{j}(\theta ,\phi )$).

Estimation involves a flow backwards from the combined information to ***θ***. Carrying out such inference in a principled manner is not straightforward and poses a number of challenges stemming from the multiplicity and the limitations in the available data sources. We illustrate the main ones below using mainly examples from recent literature on influenza, pointing out relevant ideas from the statistical literature that could be explored to address these challenges. Although, in principle, this type of synthesis can be carried out via maximum likelihood methods (e.g. Commenges and Hejblum, 2013), we mainly concentrate on a Bayesian approach as it represents a very natural approach to data assimilation both from a principled and computational point of view.

## 1 How should evidence be weighted?

When a multiplicity of data is used, the various sources of evidence will inevitably be of different quality and a natural question is whether and how to account for this diversity in the model (Ypma et al., 2012). Clearly the first challenge is to define ‘quality’. Here ‘quality’ relates to both measurement error and bias. One immediate solution to the heterogeneity of quality would be to exclude the lower quality data with, however, a resulting loss of information and risk of introducing biases due to the selective nature of information retained (Turner et al., 2009). Alternatively, a few ways of weighting data can be explored, each posing its own challenges.

The most natural approach is through an appropriate choice of distributional assumption for each data item. For example, when analysing count data, contrast the use of a negative binomial likelihood with the Poisson, as was employed in two of the transmission models developed to estimate the evolution of the 2009 A/H1N1 influenza pandemic (Birrell et al., 2011; Dorigatti et al., 2012). Dorigatti et al. (2012), in particular, demonstrate the sensitivity of estimates of *R*_0_ to the assumption of over-dispersion in the data. Furthermore, even within a specific distributional form, the degree to which error variance is modelled can have an impact upon the relative importance of each data component. This aspect of weighting of information is very closely linked to Section “Model criticism”, as the correct assumption can be examined through methods for model choice.

A further approach is to recognise and model explicitly the limitations in the data, in particular in relation to bias (e.g. see recent criticism of Google ‘Flu Trends by Olson et al., 2013). The observational model can be expanded to include additional parameters formally expressing such limitations. Magnitude and direction of the likely bias are incorporated through a suitable choice of a prior distribution for a bias parameter (Turner et al., 2009). This distribution ideally should be informative, at least in terms of the direction of the bias, to prevent the new parameter from absorbing all the unexplained variability, without offering any specific explanation for the nature of the bias. However, much remains to be done in terms of bias modelling, in particular in relation to self-reported data or data collected through particular channels, such as the Internet.

The concept of power priors (Chen and Ibrahim, 2000) represents an additional interesting avenue to be explored in the problem of weighting evidence. The principle comes from the world of clinical trials and has been proposed as an approach to incorporate data from a previous trial as an input to the analysis of a current study. The same concept could be applied to concurrent data sources, and the choice of appropriate values for the weighting scheme would be driven by expert opinion on the validity of each source or, perhaps, estimated, although this is still controversial (Neuenschwander et al., 2009).

General recommendations for the best strategy for the weighting of information do not exist, but formal thinking on how to approach such weighting of data should be encouraged as it is a choice to which modelling outcomes are rarely robust.

## 2 Handling dependence between datasets

In most cases where a multiplicity of datasets are used to inform a model, there will be some degree of dependency between them. Given a model, the important distinction is between datasets that are conditionally independent and those that are conditionally dependent. In the directed acyclic graph (Lauritzen, 1996) in Fig. 1, the datasets *x*_*j*_, *j* = 1, …, *n* are independent, conditional on the model parameters ***θ***, where the independence is represented by the lack of links between the *x*_*j*_s. This conditional independence is a common model assumption in many examples (e.g. Rasmussen et al., 2011; Strelioff et al., 2013). However, there might be situations in which the independence assumption is not tenable. An example of such data can be found in the surveillance of the 2009 influenza pandemic in the UK. Two transmission models (Birrell et al., 2011; Dorigatti et al., 2013) used, amongst other data sources, data on individuals consulting general practitioners (GPs) for influenza-like-illness (ILI). An additional relevant data source was the National Pandemic 'Flu Service (NPFS) (Evans et al., 2011), an internet and telephone service for the recording of self-reported symptoms and anti-viral distribution. It is possible that individuals contacted both their GP and the NPFS, but no information was available to identify the degree of overlap between the two datasets. On the other hand, it is reasonable to assume that appearance in one dataset is negatively correlated with appearance in the other, as the NPFS was introduced to relieve pressure on GPs. The difficulty in understanding the relationship between the two sources is the reason why the limited number of studies using GP and NPFS data (Evans et al., 2011; Brooks-Pollock et al., 2011) have made the simplifying assumption of independence. Other datasets that could potentially be informative about epidemic patterns (e.g. absenteeism, Drumright et al., 2013; Google searches, Olson et al., 2013) have so far been analysed in isolation from more traditional surveillance sources, again due to the complexity of correctly characterising the nature of this dependence.

The challenge, in this case, is both to better understand the overlap of data sources of the kind described above and to find ways of describing the resulting dependence (and likely biases) in a relevant way, even in the absence of explicit data on the overlap. This could be achieved through covariance matrices and latent variables or mixture modelling using appropriate classes of random effects distributions resorting, perhaps, to new inferential methodology. Tom et al. (2010) provide an example of this in the analysis of influenza A genomic data.

## 3 Efficient estimation of complex models

The last 20 years have seen a great progress in inferential approaches to infectious disease dynamics data (O’Neill, 2010, and references therein). Markov chain Monte Carlo (MCMC) sampling, coupled with data augmentation, have provided an unprecedented ability to tackle new problems, becoming in many ways the ‘gold standard’. However, as models acquire realism and, therefore, increase in complexity, as illustrated in the previous sections, MCMC breaks down in a number of ways. Firstly, while a likelihood might be implicit in the formulation of the model, the task of writing it down in a closed form may become impractical or impossible (McKinley et al., 2009). Secondly, the level of data augmentation required may involve imputation of more unknowns than is currently feasible to handle (Ferguson et al., 2006). Thirdly, the computational effort involved in implementing the model, for instance, to ensure convergence of the algorithm, might be prohibitive, if the ambition is to run the model in a realistic time frame (Dukic et al., 2012), while also attempting to assess model adequacy.

These problems have already emerged in the integration of phylogenetic models with more traditional transmission models (e.g. Rasmussen et al., 2011; Dearlove and Wilson, 2013); the combination of transmission dynamics with social processes (Manfredi and D’Onofrio, 2013); and the joint modelling of components of the influenza A genome over time (Tom et al., 2010).

Use of alternative Monte Carlo methods, including sequential Monte Carlo (e.g. Del Moral et al., 2006), Approximate Bayesian Computation (Marjoram et al., 2003; Toni et al., 2009) and emulation (e.g. Liu and West, 2009), either individually or in combination with MCMC, has allowed a start in tackling efficient estimation of complex models, with approximate methods of inference taking a central role. Application of these methodologies in the area of infection diseases is, however, still limited and much work is to be done to popularise them.

In the meantime, challenges continue to emerge as increasing availability of ‘big data’ (e.g. sequence data) keeps moving the goalpost. ‘Big data’ typically demand complex models. One solution is then to partition data and analyse each partition independently (Rambaut et al., 2008), ignoring any correlation. A second is to build a joint model that needs tackling with new computational methods (Tom et al., 2010) (see Section “Handling dependence between datasets”). A sensible alternative approach to the complex models that ‘big data’ might require is to proceed in steps, analysing sub-models separately first, before combining them. Different strategies for combining models exist, some of which allow feedback between different sub-models and some which do not. Work on understanding how to combine models efficiently, while still allowing for feedback where appropriate, is ongoing in evidence synthesis of other types of data (e.g. Lunn et al., 2013) and could be usefully adapted to the context of infectious disease models.

The challenge here is that existing models and inferential tools are becoming inadequate to address the demands posed by the new data paradigms.

## 4 Model criticism

Model criticism is central to any statistical analysis and particularly so in infectious disease modelling. Models are used for policy decisions and model transparency is a crucial requirement. However, model assessment is already challenging when only one source of data is involved (e.g. see Knock and O’Neill, 2014; Lau et al., 2014, for examples in the infectious diseases literature), and becomes even more problematic when simultaneously modelling multiple sources of information. Understanding identifiability, detecting and measuring conflict between evidence from the different sources and the influence of each data item on the final results are the main, interlinked, issues.

### Identifiability

In the work on transmission of the 2009 pandemic in the UK (Baguelin et al., 2010; Birrell et al., 2011; Dorigatti et al., 2013), at least three out of four available data items (data or prior information on serological testing, GP consultations, virological testing and reporting/ascertainment probabilities) were required to enable estimation of both the timing and the scale of epidemic. Estimation of the pandemic's severity (Presanis et al., 2011) had similar evidence requirements for identifiability of the case-fatality risk. In each study, understanding which items of data were crucial for identifiability of relevant quantities was only carried out informally. However, systematic understanding of identifiability and whether some parameters are only partially identified is a key step towards optimally directing resources to collection of further relevant data. Formal value-of-information methods (e.g. Fenwick et al., 2008), adapting cost-effectiveness methods to the identification of future research/information needs, have so far had limited use in the infectious disease literature. A key challenge would be to employ such methods in preparedness for future epidemics, for instance.

### Conflict

In each of the above cited studies (Baguelin et al., 2010; Birrell et al., 2011; Dorigatti et al., 2013) a number of models to reconcile the information provided by serological testing and GP surveillance data were formulated, varying from a single “rescaling factor” (Baguelin et al., 2010), through to an explicit formulation of age- and time-specific reporting rates (Birrell et al., 2011; Presanis et al., 2011; Dorigatti et al., 2013). In practice, evidence of conflict between the two sources and the potential for unaccounted sampling and ascertainment biases in both the serological testing data (Miller et al., 2010, and response) and clinical case estimates (Evans et al., 2011) was explored through sensitivity analyses explicitly modelling the biases in Presanis et al. (2011).

The possibility of multiple sources of data, depending on shared parameters, to provide conflicting inference poses a number of challenges in model criticism. There are different ways to define conflict (or consistency) and an expanding area of research concerns methods to detect and measure such conflict (e.g. Presanis et al., 2013, and references therein). Having identified conflicting evidence, the next step is to pinpoint the cause of the inconsistency, e.g. whether the data have been misinterpreted or biases not properly acknowledged and hence to reconcile the differences. Conflict is a property of a network of evidence, not of a single data source, so whether it is possible to identify *which* data sources may be responsible for the conflict (e.g. biased) may be context-dependent. There are many possibilities for resolution (e.g. weighting evidence by accounting for bias, see Section “How should evidence be weighted?”), that may lead to different inferences, and hence to the problem of model choice. Deciding on the best strategies for detecting, measuring and resolving conflict is a key future challenge.

### Influence

Highly related to the assessment of conflict and weighting of evidence is the assessment of how influential is each item of evidence and/or model assumption. Each of the various studies on the 2009 pandemic showed that inferences could be sensitive to different model assumptions. However, formal methods for quantifying the influence of different data sources in the context of infectious disease modelling are not as widely used as in traditional contexts (e.g. residual and influence analysis in regression) or in other fields (e.g. geo-physical science or economics, see Saltelli et al., 2000) although examples of formal sensitivity analyses (e.g. cross-validation) have started to appear (Ypma et al., 2012).

Again, the challenge here resides in adapting available methodologies to epidemic models for which standards do not yet exist.

## Discussion

In conclusion, we have argued that the epidemic models needed to answer policy questions can seldom be informed by a single source of information, and that the favourable scenario of a unique dataset for any given model parameter is unrealistic. This raises a set of significant challenges. Meeting them will require more thoughtful model formulation, better exploitation of currently available statistical tools and perhaps the development of new (most likely approximate) methods of inference. These efforts will, however, result in significant improvements in terms of defensibility of epidemic models. Also, the significant challenges posed by the epidemic context offer the opportunity to contribute to general development of statistical methodology.
